# Climate change and health in Bangladesh: a baseline cross-sectional survey

**DOI:** 10.3402/gha.v9.29609

**Published:** 2016-04-04

**Authors:** Md Iqbal Kabir, Md Bayzidur Rahman, Wayne Smith, Mirza Afreen Fatima Lusha, Abul Hasnat Milton

**Affiliations:** 1 Centre for Clinical Epidemiology and Biostatistics, School of Medicine and Public Health, Faculty of Health and Medicine, The University of Newcastle, Callaghan, NSW, Australia; 2 National Institute of Preventive and Social Medicine, NIPSOM, Dhaka, Bangladesh; 3 Climate Change and Health Promotion Unit, Ministry of Health and Family Welfare, Dhaka, Bangladesh; 4 School of Public Health and Community Medicine, Faculty of Medicine, UNSW, Sydney, Australia; 5 Health Communication Network, Dhaka, Bangladesh

**Keywords:** climate change, health, adaptation, household, vulnerable community, Bangladesh

## Abstract

**Background:**

Bangladesh is facing the unavoidable challenge of adaptation to climate change. However, very little is known in relation to climate change and health. This article provides information on potential climate change impact on health, magnitude of climate-sensitive diseases, and baseline scenarios of health systems to climate variability and change.

**Design:**

A cross-sectional study using multistage cluster sampling framework was conducted in 2012 among 6,720 households of 224 rural villages in seven vulnerable districts of Bangladesh. Information was obtained from head of the households using a pretested, interviewer-administered, structured questionnaire. A total of 6,720 individuals participated in the study with written, informed consent.

**Results:**

The majority of the respondents were from the low-income vulnerable group (60% farmers or day labourers) with an average of 30 years’ stay in their locality. Most of them (96%) had faced extreme weather events, 45% of people had become homeless and displaced for a mean duration of 38 days in the past 10 years. Almost all of the respondents (97.8%) believe that health care expenditure increased after the extreme weather events. Mean annual total health care expenditure was 6,555 Bangladeshi Taka (BDT) (1 USD=77 BDT in 2015) and exclusively out of pocket of the respondents. Incidence of dengue was 1.29 (95% CI 0.65–2.56) and malaria 13.86 (95% CI 6.00–32.01) per 1,000 adult population for 12 months preceding the data collection. Incidence of diarrhoea and pneumonia among under-five children of the households for the preceding month was 10.3% (95% CI 9.16–11.66) and 7.3% (95% CI 6.35–8.46), respectively.

**Conclusions:**

The findings of this survey indicate that climate change has a potential adverse impact on human health in Bangladesh. The magnitude of malaria, dengue, childhood diarrhoea, and pneumonia was high among the vulnerable communities. Community-based adaptation strategy for health could be beneficial to minimise climate change attributed health burden of Bangladesh.

## Introduction

Health risk attributed to climate change is a multidimensional and cross-cutting issue (1, 2). The Fifth Assessment Report of the Intergovernmental Panel on Climate Change (IPCC) reinforced adaptation needs to protect human health from the adverse consequences of climate change (3). Community-based strategic interventions will be needed for health adaptation in developing countries such as Bangladesh over the coming years (4–7).

However, climate change and health related studies are mainly reported in developed countries and the number of studies in low-income countries are very limited (8–14). Since 2007, Bangladesh has topped the IPCC's risk index for climate change (15), which directly affects the lives and livelihoods of 36 million people in the southern coastal regions (16). Millions of people in Bangladesh suffer directly or indirectly from water and vector-borne diseases such as diarrhoea, malaria, and dengue (17, 18). The actual health impacts of climate change are likely to be influenced by local environmental conditions, socioeconomic circumstances, and behavioural adaptations taken to reduce the full range of threats to health (19–22).

The risk to human health in Bangladesh is one of the major risks arising from climate change. Incremental changes in average climate variables and climate variability – in particular, extreme weather events – affect disease outcomes. Due to climate change–induced differences in temperature and precipitation, the dynamics of vector-borne diseases such as dengue, malaria, and so on, will change (23). Meteorological factors are significantly associated with the risk of childhood diarrhoea in rural Bangladesh (24). The health sector must have evidence-based strategies for adaptation to new patterns of infectious disease under climate change (25). Therefore, studies focused on climate change and its potential health effects are most warranted in Bangladesh. However, little work has been done to quantify the magnitude of climate change effect on health in Bangladesh. In a cross-sectional study from two villages, one from the northern and another from the southern part of Bangladesh, households’ (*n*=450) perception and human health risks were explored, which warrants further research with a larger sample size for generalization (26).

In the absence of valid surveillance data, a prospective cohort study is suitable to determine the changing disease patterns and the increased risk of certain diseases due to climate change. Hence, it may take a longer time to establish an association between increased risk of specific diseases and climate change. We conducted this cross-sectional survey as the basis of a future cohort study.

The survey was jointly conducted by the Climate Change and Health Promotion Unit (CCHPU) of the Ministry of Health and Family Welfare Bangladesh, and the Health Communication Network and the University of Newcastle, Australia. The objective of this study was to collect information on vulnerable peoples’ experience regarding potential influence of climate change and effect of extreme weather events on health, the magnitude of climate-sensitive diseases, and scenarios of the impact of climate variability and change on health system.

The health system of Bangladesh is publicly financed by a top-down approach from national to community level. There is no health insurance or community financing for health care expenditure. In most cases, the poor population pays out of pocket for their health care. This study also assessed availability and accessibility of health system at community level for adaptation. However, the knowledge and perception portion of the study has been submitted elsewhere.

## Methods

### Study population

This cross-sectional study was conducted between July and September 2012 in seven districts of Bangladesh that are prone to cyclone, flood, and salinity (23). The targeted study population was the total community of the vulnerable seven districts, that is, Bagerhat, Barguna, Cox's Bazar, Faridpur, Khulna, Satkhira, and Sirajganj comprising a population of 19,228,598. Multistage cluster sampling was used for recruiting the study population. We randomly selected four upazilas (sub-districts) from each district, resulting in a total of 28 upazilas as the primary sampling units. Then, four unions (lowest administrative unit) per upazila were selected randomly, resulting in a total of 112 unions. Finally, two villages from each union were selected randomly, reaching a total of 224 villages. From each selected village, we randomly selected 30 households using the Bangladesh Bureau of Statistics (BBS) household numbers. Therefore, a total of 6,720 households were surveyed in rural areas. From each household, we selected the head of the household as an eligible adult participant providing written consent or thumb impression; in the absence of the head of the household (mostly male), we interviewed the next seniormost female household member as the respondent. Information on other household members was collected from the respondent.

### Data collection

After obtaining written informed consent, information was collected using a pretested, structured, interviewer-administered questionnaire. The questionnaire was developed by the investigators and finalized after pretesting in a similar rural setting outside the study area. A week-long training was provided to the interview team prior to data collection. The trained 28 interviewers collected data through door-to-door visits at household level under the direct supervision of 14 field supervisors. The interviewers and supervisors had completed bachelor degrees and had experience in conducting national-level surveys using the census method. The quality control team formed by the investigators monitored the performance of field members through regular observation at the household level and regular checks of data for completeness. In 5% of study participants (*n*=336), the quality control team independently repeated data collection. The investigators checked each questionnaire to ensure that no information was missing; any error detected was corrected immediately at the field.

We have also collected secondary data of weather variables (rainfall, average temperature, maximum temperature, minimum temperature, and relative humidity) since 1970 to 2010 from the Bangladesh Meteorological Department (BMD).

### Interview questions

In addition to socio-demographic variables (family size, education, occupation, source of drinking water, total family income, duration of stay, etc.) and climate-related variables, we collected household data on malaria, dengue, pneumonia, and diarrhoeal diseases from every member of the household. Height and weight of the respondents were measured to obtain body mass index (BMI) for nutritional assessment. Other relevant information on primary health care facilities, school health promotion, and health system response were also collected through face to face interview.

Dengue and malaria were self-reported by survey participants with a review of written history of diagnosis by a general practitioner or village doctor. In extreme weather events (flood, cyclone, storm surge, and drought), snakes lose their normal habitat and come out from their hideouts and move towards the households. Therefore, incidence of morbidity and mortality from snake bites increases during extreme weather events. We have compared our findings with the national-level household survey data of snake bites. The number of snake bites received by any household member for the past 12 months was recorded. Death from drowning increases during flood, cyclone, storm surge, and so on, which are related to extreme weather events. Mortality from drowning and snake bites is an important health indicator during extreme weather events. The number of deaths from drowning and snake bites during extreme weather events was quantified for the past 10 years preceding the interview. Household incidence of diarrhoea and pneumonia in children aged under-5 years was collected as number of episodes (at least one) for the last 12 months and for the last 1 month preceding the interview date. One episode of diarrhoea was defined as acute watery stool passed more than three times in 24 h or diagnosed by a registered physician. One episode of pneumonia (acute respiratory illness) was defined as fever, running nose, and/or cough, with shortness of breath and increased respirations (in drawing of ribs on observation) or diagnosed by a registered physician.

Regarding the diseases, respondents were asked about total family members’ outcome inclusive of his/her own. Female members of the family contributed to the response by reporting children's disease outcomes.

### Statistical analysis

Collected data were initially entered into computer using the Statistical Package for Social Sciences (SPSS), version 21. Data were validated by a series of logical and range checks, producing summary statistics and tables. Data were immediately copied onto the hard disks of two computers as soon as data verification was completed and a copy was sent to the principal investigator at the University of Newcastle, Australia. Data were then transferred and analysed using STATA 13.

The summary statistics were reported as means, with standard deviations (SD) for continuous variables or percentages, with 95% confidence intervals (CI) for categorical variables. Socio-demographic variables and participants’ responses were summarized and presented using frequency tables. We estimated the incidence of climate-sensitive diseases (e.g. dengue, malaria) by fitting intercept only Poisson model using total number of household members as the offset. We estimated the incidence of different health events (e.g. diarrhoea) among under-five children adopting the same methods as described above using the total number of under-five children in each house as the offset. To adjust for the design effect of multistage sampling, we created sampling weights and incorporated them in the analyses using ‘svy’ command in Stata for all the analyses. We calculated sampling weights in four stages (i.e. the selection of upazila, union, village, and household) taking the inverse of the sampling fraction. The base weight was calculated by multiplying weights from all the four stages and was used in the ‘svy’ command.

### Ethical approval and consent

The study protocol was approved by the Bangladesh Medical Research Council and Human Research Ethics Committee of the University of Newcastle, Australia (H2012-0163). Participants were provided with information note about the study before obtaining written consent.

## Results

Among the 6,720 respondents, 92.9% were males as head of the households and the mean duration of their stay in that particular area was 30 years (median+ 25, SD+15). Table 1 presents the socio-demographic characteristics of the participants. The majority were day labourers or farmers (60%), and almost 90% earned a monthly income of below 12,000 Bangladeshi Taka (BDT) (156 US$ approx. in 2015). As per the BMI categories for developing countries, about 22% of the participants were underweight, almost 22% were overweight, and 4% were obese (Table 1).

**Table 1:** Socio-demographic characteristics of the participants (*n*=6,720)

Variable	Frequency (%)
Gender (male)	6,245 (92.9)
Age (mean ±SD) in years	44.7±13.5
Duration of stay in this locality (mean, median, SD) in years	30, 25, ±15
Education of respondent	
No formal education	2,923 (43.5)
Primary	2,013 (30.0)
Secondary	1,143 (17.0)
Higher secondary	496 (7.4)
Graduate and above	145 (2.2)
Occupation of respondent	
Farmer	1,988 (29.6)
Day labourer	2,047 (30.5)
Service holder	524 (7.8)
Small and medium business	1,066 (15.9)
House wife	299 (4.5)
Fisherman	252 (3.8)
Unemployed	51 (0.8)
Others	493 (7.3)
Total household monthly income	
Income (1 US$=77 BDT in 2015 approx.)	
<4,000 BDT	1,436 (21.4)
4,000–8,000 BDT	3,485 (51.9)
8,000–12,000 BDT	1,127(16.7)
>12,000 BDT	672(10)
Drinking water source	
Shallow tube well	2,936 (43.7)
Deep tube well	2,822 (42.0)
Supply water	36 (0.5)
Untreated water	322 (4.8)
Treated water	524 (7.8)
Rain water	50 (0.7)
Others	30 (0.5)
Nutritional status (BMI, weight in kg/height in m^2^)	
Underweight (<18.5)	1,469 (21.9)
Normal weight (18.5 to <23)^a^	2,545 (52.7)
Over weight (23 to <27.5)	1,443 (21.5)
Obese (≥27.5)	263 (3.9)

a Developing country cut off range of BMI.

BDT=Bangladeshi Taka; BMI=body mass index; SD=standard deviation.

The self-reported effect of climate variability and extreme weather events on livelihood was analysed. Most of the respondents (96%) faced extreme weather events in their locality. In the past 10 years, almost half of the respondents (45.2%) had been homeless for more than a month (mean 38 days) because of floods and cyclones. Among the homeless, about 40% were displaced twice and 20.5% more than twice. Regarding agricultural impact on nutrition, almost 71% believed that food crop production reduced during the past decade from their field experiences (Table 2).

**Table 2:** Effect of extreme weather events on livelihood (*n*=6,720)

Variable	Frequency (%)
Have you ever faced any extreme weather event during your stay in this locality?	
Yes	6,434 (95.7)
Type of extreme weather events faced in this area^a^	
Flood	4,908 (76.3)
Cyclone	5,139 (79.8)
Tidal wave	3,234 (50.3)
Drought	3,134 (48.7)
River bank erosion	931 (14.5)
Earth quake and others	11 (0.2)
Did you become homeless due to extreme weather events in the past 10 years?	
Yes	2,912 (45.2)
Frequency of homelessness in past 10 years	
Once	1,161 (39.9)
Twice	1,154 (39.6)
More than two times	598 (20.5)
Number of homeless days in the past 10 years due to extreme weather events (mean, median, range, SD)	38, 5, 1–750, ±111
Food crop production reduced in the past 10 years	4,761 (70.8)

a Percentage total may add up to more than 100% as multiple responses were permissible. SD=standard deviation.

Health problems, health– climate change links, and contextual issues like health care access, expenditures, and poverty were also reported. Regarding health safety, deaths from drowning 116 (1.7%) and snake bite 26 (0.4%) during the extreme weather events in the past 10 years were substantial. Number of snake bites (does not include deaths) in the past 12 months was 207 (3.1%). Non-availability of qualified service providers, such as community clinics (28%) and union sub-centres (25.1%), was higher in remote settings in comparison with upazila (15.8%) and district levels (11.1%). Almost all of the respondents (97.8%) believed that health care expenditure increased after the extreme weather events. Mean annual total health care expenditure was 6,555 BDT (1 USD=77 BDT in 2015 approx) which was 8.3% of the mean annual income (Table 3).

**Table 3:** Health problems and contextual issues of health in vulnerable community (*n*=6,720)

Variable	Frequency (%)
Death from drowning in the past 10 years	116 (1.7)
Death from snake bite in the past 10 years	26 (0.4)
Availability of service providers at nearby health facility when necessary	5,211 (77.6)
Non-availability of service providers when necessary	
District hospital	37 (11.1)
Upazila health complex	319 (15.8)
Union health and family welfare centre	615 (25.1)
Community clinic	538 (28.0)
Increased health care expenditure after extreme weather events	6,572 (97.8)
Total health expenditure for family in the past 12 months in BDT (mean, median, range, SD)	6,555, 3,800, 100–150,000, ±9,581
Total health expenditure for all households (6,555×6,720) BDT	44,049,600

BDT=Bangladeshi Taka; SD=Standard deviation.

**Fig. 1:**
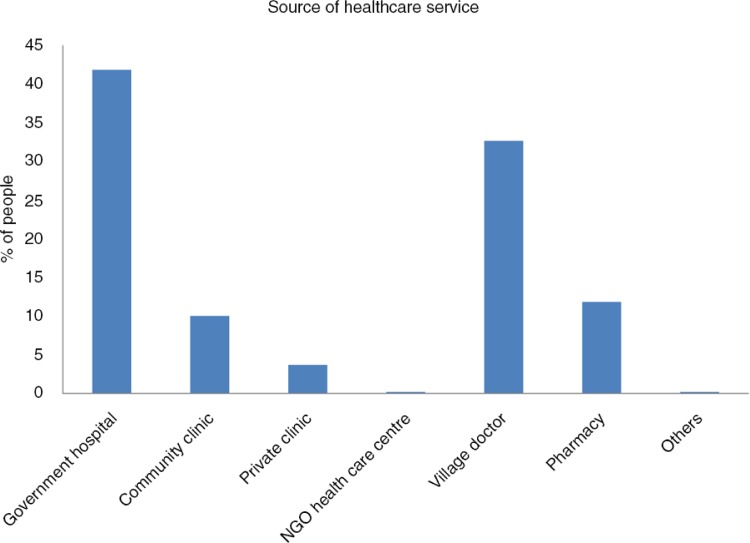
Source of healthcare services received by the respondents following extreme weather events.

**Fig. 2:**
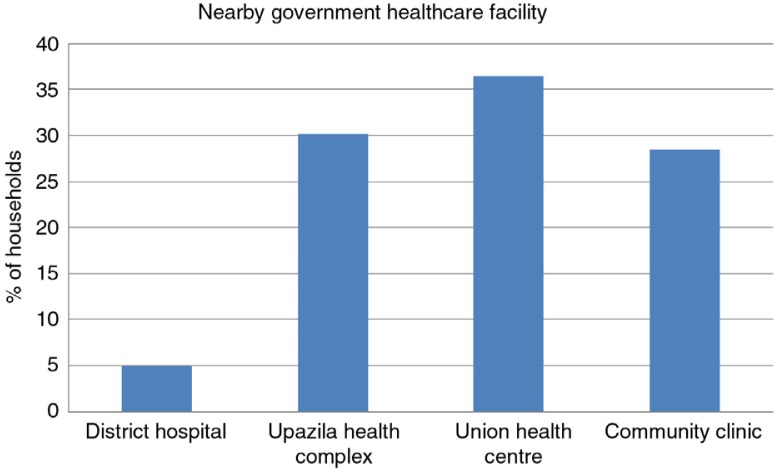
Nearby government healthcare facility within 2 km radius of the household.

About 52% of the participants got health care services from government facilities that includes 10% services received from village-level community clinics (Fig. 1). A substantial number of households got the service from unqualified providers, either village doctors (33%) or local drug stores (12%). About 65% of households were nearby (2 km radius) to remote rural government health care settings such as community clinics (28.5%) and union health centre (36.5%) (Fig. 2).

The estimated result of incidence of dengue was 1.29 (95% CI 0.65–2.56) and malaria was 13.86 (95% CI 6.00–32.01) per 1,000 adult population per year. The preceding month data show the incidence of diarrhoea and pneumonia among under-five children to be 10.3% (95% CI 9.1–11.6) and 7.3% (95% CI 6.3–8.4), respectively (Table 4).

**Table 4:** Incidence of climate-sensitive diseases and health events in the households

Outcome indicator	No. of persons (%)	Incidence per 100,000 (95% CI)
Any family member suffered from dengue in the past 12 months	34 (0.51)	122 (88–168)
Any family member suffered from malaria in the past 12 months	295 (4.4)	1,199 (1,082–1,329)
Any family member suffered from snake bite in the past 12 months	207 (3.1)	36 (29–45)
Any children <5 years suffered from diarrhoea in the past 12 months^a^	700 (31.4)	
Any children <5 years suffered from pneumonia in the past 12 months^a^	529 (23.8)	
Any children <5 years suffered from diarrhoea in the past 1 month^a^	253 (11.4)	8.3 (7.4–9.4)^b^
Any children <5 years suffered from pneumonia in the past 1 month^a^	182 (8.2)	8.5 (7.4–9.8)^b^

a At least one episode.

b Incidence rate in per cent.

## Discussion

Our study is the first large-scale quantitative attempt to assess the impact of climate change on health of the vulnerable communities in Bangladesh (27). Similar studies have been conducted in Philippines (28), Vietnam (29), the United States of America, Canada, Malta (30), Nigeria (31), Nepal (32), India (33), Australia (34), and China (35), although their main focus was on people's perception about the impact of climate change on health. A cross-sectional study of two villages in Bangladesh had some limitations with regard to generalizability but their findings on climate-related health coping supports some of the findings of our study (26). The average total health expenditure was more in our study (6,555 BDT) when compared with the study by Haque et al. (17) (2,250 BDT). Our sample size was larger, representative, and geographically more well-distributed among vulnerable communities when compared with studies in other developing countries, such as Nepal and Trinidad and Tobago (36, 37). This survey revealed that the vulnerable community believes that the climate is changing with an effect on human health in Bangladesh. This information is supported by the BMD data from 1951 to 2011, SAARC Meteorological Research Centre (SMRC), and other studies (38–44).

Our study finding showed that almost half of the families (approximately 9 million people in the coastal catchment) became homeless and displaced for a long time due to extreme weather events such as floods, cyclones, and so on, with a mean of 38 days. Almost one-third of the families experienced displacement twice in a span of 10 years. According to the Internal Displacement Monitoring Centre, more than 42 million people were displaced in Asia and the Pacific during 2010 and 2011 (45). Norman Myers, a British environmentalist, predicted that, in 2050, 15 million people will be displaced in Bangladesh (46).

This survey has focused some light on the recent initiative of the Bangladesh government (2009) to decentralize health care facilities up to the village level through community clinics (for every 6,000 of the rural population). There are four tiers of health care service delivery in the government setup of Bangladesh: community clinics (6,000 rural population at the village level), Union Health and Family Welfare Centres (at the union level), Upazila health complex (50-bedded hospitals at the sub-district level), district hospitals (100–250 bedded) and tertiary-level hospitals (medical colleges and specialized hospitals). We tried to explore which facilities were most accessible to the vulnerable community and to what extent. We also explored the services obtained from unqualified service providers. This is not directly related to climate change but there is an indirect correlation. During extreme weather events (such as floods) the communication is disrupted and accessibility is hampered (26).

The annual total health expenditure estimate of all households was tremendous (approximately 44 million BDT) and coming from OOP of the low-income climate vulnerable group (47, 48), which was even higher than the estimate of Bangladesh National Health Accounts (BNHA) 1997–2012 (49). This could be useful for the Health Economics Unit (HEU) of the Ministry of Health and Family Welfare (MOHFW) to calculate the direct health expenditure of the climate-vulnerable communities of Bangladesh in NHA.

Our study area was outside of the current endemic districts of malaria; still 295 self-reported cases of malaria were found in the non-endemic areas. This finding supports the prediction of the ‘Second National Communication of Bangladesh to the UNFCCC’ that the malaria-affected areas will increase in future and some new areas will be exposed to it (50). The number of deaths from drowning and snake bites corroborates with other community-based studies in Bangladesh (51, 52). During extreme weather events, such as floods and cyclones, the normal habitat of snakes are lost and they come out in the community which accounts for the increased number of snake bites. So, there is again an indirect relationship of the increased number of extreme events with that of the increased number of snakebites and drowning cases as well.

In our study, participants strongly agreed that agricultural food crop production had reduced from 10 years back. Nutritional status is affected by food crop production and there might be a correlation with climatic variability which needs to be explored by further study (53, 54). As most of the respondents were agro-based by occupation (mostly farmers), this is a general observation from their field experiences. We did not explore the link of climate change in this study, but the impact on nutrition (as from the BMI) could be explored in future by linking these information. The nutritional status showed that almost half of the respondents were beyond normal weight. This finding is important for local-level planning and may help policy makers to develop and implement effective adaptation measures.

Regarding childhood diarrhoea, our study is supported by the findings of International Centre for Diarrhoeal Diseases Research, Bangladesh (ICDDRB) Matlab 2000–2006 study (24). The Bangladesh Demographic and Health Survey (DHS) shows 5% of diarrhoea and 6% of pneumonia (Acute Respiratory Illness, ARI) 2 weeks preceding the survey in 2011, respectively (55). A direct comparison of disease incidence by month and season with the 2011 DHS is complicated because of location effect of primary sampling units. Our study was conducted at highly vulnerable locations and during the post-monsoon season. That might be the cause of the high incidence rate of diarrhoea and pneumonia in children aged under 5 years. ICDDRB studies reported that climate variability is strongly linked with the incidence of childhood illnesses and that the impact of climate varies by season and location (56, 57). One study showed that, in tropical and subtropical regions, high precipitation may cause the outbreak of diarrhoea (58).

There were a few limitations to this study, including the recall bias, as we relied on the previous experiences and subjective judgements of the head of the households. However, a similar approach was adopted in several other studies (10, 32, 34, 59). Self-reported vector-borne diseases such as malaria and dengue were not confirmed with the support of laboratory reports; self-reported diseases might not be the same as physician-based diagnoses. Such data could be collected in future and compared for assessment of the accuracy of community perceptions. As 92.9% of participants were male, this could be an important limitation with regard to female evaluation of climate–health links. However, in the Bangladeshi context, as in the rest of South-East Asia, males are predominantly ‘the head of the household’ and females often provide household information through their male partners.

The strengths of the study, however, outweigh the few limitations. The study recruited participants from an array of geographic locations known for their vulnerability to climate change. A large number of resource-poor rural households along the coast line and other vulnerable areas had been surveyed. This makes the findings from this study relevant to other climate-vulnerable areas of Bangladesh and South-East Asia as well (Ganges delta). A further strength is the collection of data from local community context with primary experiences of extreme weather events. The findings will provide strategic directions for sectoral policy implication of Health Population and Nutritional Sector Development Programme (HPNSDP) of Bangladesh. In addition, this study will be useful to formulate Behaviour Change Communication (BCC) activities at individual and community level for adaptation to health. The findings could be used as a baseline for a future cohort study.

## Conclusions

This study is pivotal in providing direction for public health adaptation to climate change in Bangladesh. It is based on rich interview material from a large number of respondents living in poor and vulnerable communities. Results illustrate how marginalized populations are at risk of suffering from climate change–related health impacts and how the country's public health system needs to be adapted to climate change in the future.

The findings of this study indicate that there is a potential risk of climate change on human health. The magnitude of malaria, dengue, childhood diarrhoea, and pneumonia is high among the vulnerable communities. Prevention and control of climate-sensitive diseases have to be addressed with area-specific interventions guided by local-level planning of the low-income vulnerable communities. Government initiatives, public–private strong advocacies, and international collaborations are needed to reduce OOP payments through alternative health care financing for climate victims. Climate change ultimately has an impact on health; therefore, public health care facilities at the community level should be prepared to be utilised to their full potential. The health system needs strategic interventions to cope with climate-sensitive health problems. Increasing the number of visits to public health facilities (10% visits to community clinics in this study) instead of to unqualified providers (45%) to cope with climate-induced health problems is a strategic challenge for policy makers. Community-based adaptation strategy for health could be beneficial for sectoral approach in ensuring sustainable development.

Further studies are warranted to confirm shifting of vector- borne diseases such as malaria from endemic to non-endemic zones. Child-centred and school-based intervention studies could be explored to reduce seasonal childhood diarrhoea, pneumonia, and malnutrition A future cohort study, based on this survey, might allow public health scientists and policy makers to take effective measures to minimise climate change attributed health burden of Bangladesh and other developing countries as well.
